# Aptamer Conjugated Indium Phosphide Quantum Dots with a Zinc Sulphide Shell as Photoluminescent Labels for *Acinetobacter baumannii*

**DOI:** 10.3390/nano11123317

**Published:** 2021-12-07

**Authors:** Zeineb Ayed, Shiana Malhotra, Garima Dobhal, Renee V. Goreham

**Affiliations:** 1 MacDiarmid Institute for Advanced Materials and Nanotechnology, Wellington 6012, New Zealand; zeineb.ayed@vuw.ac.nz; 2 School of Chemical Physical Sciences, Victoria University of Wellington, Kelburn, Wellington 6012, New Zealand; 3 School of Mathematical and Physical Sciences, University of Newcastle, Newcastle, NSW 2308, Australia; shiana.malhotra@uon.edu.au (S.M.); gsd534@newcastle.edu.au (G.D.)

**Keywords:** quantum dots, aptamers, *Acinetobacter baumannii*, photoluminescent labels, fluorescence

## Abstract

*Acinetobacter baumannii* is a remarkable microorganism known for its diversity of habitat and its multi-drug resistance, resulting in hard-to-treat infections. Thus, a sensitive method for the identification and detection of *Acinetobacter baumannii* is vital. However, current methods used for the detection of pathogens have not improved in the past decades and suffer from long process times and low detection limits. A cheap, quick, and easy detection mechanism is needed. In this work, we successfully prepared indium phosphide quantum dots with a zinc sulphide shell, conjugated to a targeting aptamer ligand, to specifically label *Acinetobacter baumannii*. The system retained both the photophysical properties of the quantum dots and the folded structure and molecular recognition function of the aptamer, therefore successfully targeting *Acinetobacter baumannii*. Confocal microscopy and transmission electron microscopy showed the fluorescent quantum dots surrounding the *Acinetobacter baumannii* cells confirming the specificity of the aptamer conjugated to indium phosphide quantum dots with a zinc sulphide shell. Controls were undertaken with a different bacteria species, showing no binding of the aptamer conjugated quantum dots. Our strategy offers a novel method to detect bacteria and engineer a scalable platform for fluorescence detection, therefore improving current methods and allowing for better treatment.

## 1. Introduction

The current state of the world and the ongoing fight against the coronavirus disease 2019 (COVID-19) demonstrates that the study of microbes is far from developed. It has become obvious that existing methods lack sensitivity and require tedious analysis [1]. While the challenges presented by viral infections are significant, a superbug (a multi-resistant bacteria) had been predicted to cause greater complications in the future. Experts from the World Health Organisation (WHO) recommend that priority should be given to carbapenem-resistant Gram-negative bacteria including *Acinetobacter baumannii* (*A. baumannii*) and *Pseudomonas aeruginosa* (*P. aeruginosa*) [2].

*A. baumannii* is a Gram-negative bacteria capable of causing both community and health-care associated infections [3,4,5]. The fatality rate related to *A. baumannii* has been reported as 61.6% [6]. The organism’s ability to survive under a wide range of environmental conditions and to persist for extended periods of time on surfaces make it a frequent cause of outbreaks [7,8,9]. It is responsible for many infections including pneumonia, meningitis, urinary tract infections and wound infections [6,10,11]. Thus, a sensitive method for the identification and the detection of *A. baumannii* is crucial to preventing the spread of infections.

Acinetobacter genus are known to be carbapenem-resistant Enterobacteriaceae, which means they possess the gene blaOXA that is associated with resistance to common antibiotics, such as cabapenem. With the use of the multiplex polymerase chain reaction (PCR), Turton et al. [12], confirmed the existence of the gene blaOXA-51-like in only *A. baumannii* making it specific to this species. Hence, a method to identify *A. baumannii* by the detection of the blaOXA-51-like primers using multiplex PCR was developed. Whether PCR can detect all the variants of the gene is still uncertain. PCR is also a very expensive method, has lower specificity and needs an extensive list of specific primes leading to additional complexity [13]. 

More recently, other identification methods that target *A. baumannii* have focused on using aptamers. Aptamers are short single strands of DNA or RNA with the ability to form a particular structure that can bind to specific target molecules [14]. Through 12 cycles of system evolution of ligands by an exponential enrichment process (SELEX), an aptamer with high binding affinity towards *A. baumannii* was selected by Rasoulinejad et al. [15], The aptamer combined with a specific antibody were then used in an enzyme-linked aptamer sorbent assay (ELASA) to detect 47 *A. baumannii* isolates [15]. Since the discovery of aptamers 10 years ago, interest has increased due to low costs and ease of use. For these reasons, aptamers have widespread applications, including environmental sensors, clinical reagents and biophysical discoveries [16]. Aptamers can also be combined with fluorescent molecules such as organic dyes and quantum dots (QDs) that can be used as fluorescent biosensors [17,18]. 

Recent studies have employed techniques using microfluidics devices or other novel sensors for the detection of single strains of bacteria (i.e., surface plasmon resonance and electrochemical impedance spectroscopy). Su et al., developed an electromagnetically driven microfluidic platform combined with dual aptamer assay for the detection of *A. baumannii* [19]. Although this method can detect the bacterium with high specificity and sensitivity within 30 min, it has some limitations. For instance, the obstacle of manufacturing a strong magnet in a limited size is an issue Moreover, the method is not sensitive enough for the detection of small bacteria concentrations. 

The use of targeting QDs offers a multimodal detection alternative by combining optical and electrochemical platforms for bioanalysis. Cadmium-based QDs have been applied in various fields such as bioimaging and bioanalysing [20]. The presence of highly toxic cadmium hinders their use in biological applications. InP/ZnS QDs possess the same physico-chemical properties as the cadmium-based QDs but different particle cores that are less toxic [21,22]. In addition, QDs are a better alternative to organic dyes because they exhibit high efficiency, high photostability and long fluorescence lifetime [23]. Aptamer-conjugated QDs have already been proven to be stable in extreme environmental conditions, which is important as *A. baumannii* inhabit soil, aquatic environments, freshwater ecosystems and wastewater treatment plants [24]. 

Herein, InP/ZnS QDs are synthesised and conjugated to a targeting aptamer (QDs-aptamer) for fast and sensitive labelling of *A. baumannii*. The specificity of the QDs-aptamer was verified using confocal microscopy and transmission electron microscopy (TEM). Our method offers an innovative strategy to detect bacteria and engineer a scalable platform for fluorescence detection. The combination of QDs and aptamers for ELISA-like methods is a novel and unique strategy that combines the specificity of the aptamer along with the fluorescence of the QDs. This method is very good for detection of colonies offering high sensitivity to low concentrations of bacteria and high stability in diverse environments. The use of photoluminescent labelling provides a novel method for bacteria detection that can be easily adapted to other targets, such as proteins, mammalian cells, and other biological entities. In addition, simple detection methods, such as ELISA can be easily adapted and used. 

## 2. Materials and Methods

### 2.1. Synthesis of InP/ZnS Quantum Dots 

InP/ZnS QDs were synthesised using a previously published procedure by Tessier et al. [25], and 0.45 mmol of indium(III)chloride and 2.2 mmol of zinc(II)chloride were first mixed in 5 mL of oleylamine and heated to 120 °C under a vacuum. After 60 min the reaction was put under a nitrogen atmosphere and heated to 180 °C. Once the desired temperature was reached, 1.6 mmol of tris-(diethylamino) phosphine was injected quickly into the mixture to allow the InP core growth. This step is considered our zero-time point (*t* = 0 min). After 20 min, 1 mL of 2.2 M sulphur in trioctylphosphine (TOP-S) was slowly injected over a period of 10 min. Subsequently at 60 min, the temperature was increased from 180 °C to 200 °C. At 120 min, 1 g of zinc stearate in 4 mL of 1-octadecene (ODE) was slowly injected dropwise over a period of 10 min, after which the temperature was increased from 200 °C to 220 °C. At 150 min, 0.7 mL of TOP-S was injected slowly over a period of 10 min after which the temperature was increased to 240 °C. 0.5 g of zinc stearate in 2 mL of ODE was slowly added at 180 min, after which the temperature was increased to 260 °C. The reaction was stopped after 210 min by cooling down the temperature to 70 °C. The InP/ZnS QDs were then diluted with toluene, precipitated in ethanol, and resuspended in toluene. Further purification was undertaken using a size exclusion column in toluene. This yielded red emitting (~600 nm) toluene soluble QDs. 

### 2.2. Ligand Exchange with Mercaptosuccinic Acid 

0.30 g of mercaptosuccinic acid (MSA) was mixed with 1 mL of toluene and stirred for 10–15 min to ensure that MSA was dissolved, Next, 1 mL of InP/ZnS QDs (20 mg/mL) were added to the mixture and allowed to stir for another minute. Finally, 1 mL of ammonium hydroxide and 1 mL of Milli-Q water were added, and the reaction was left to stir for 5 h or until all the QDs migrated to the water phase. The coloured aqueous layer was collected and purified by precipitation in ethanol and centrifugation. The supernatant was discarded, and the pellet was redispersed in 1 mL of Milli-Q water. 

### 2.3. Conjugation of the InP/ZnS QDs-MSA with Aptamers 

100 µL of freshly prepared N-hydroxysuccinimide (0.05 M) and 1-ethyl-3-(3-dimethylaminopropyl) carbodiimide (0.02 M) were mixed with 1 mL of 2 mg/mL QDs-MSA for 10 min. We added 10 µL of the amino-functionalised aptamer (0.1 mg/mL) to the solution and further stirred it for 2–3 h. The as prepared QDs-aptamer conjugate solution was washed using a 30 kDa Amicon centrifugal filter Units (Merck, Darmstadt, Germany) with water after which it was resuspended in 1 mL of Milli-Q water. 

### 2.4. Bacterial Culture and QDs-Aptamer Coupling

Both of the *A. baumannii* strains used in this work (AB9 and AB11) were multidrug resistant isolates received from Dr. Matthew Blakiston from LabPlus Microbiology Department Auckland City Hospital, New Zealand. Another bacterial strain used for *P. aeruginosa* is PA01 (ATCC 15692) obtained from Environmental Science and Research Culture Collection (Porirua, New Zealand).

At the mid-log phase *A. baumannii* and *P. aeruginosa* solutions were centrifuged for 10 min at 2000× *g*. The supernatant was discarded, and the pellet was washed three times by centrifugation and resuspension in wash buffer containing phosphate-buffered saline (PBS) and 0.02% polysorbate 20 (Tween 20). After washing, the pellet was resuspended in 200 µL of binding buffer containing wash buffer and 1% bovine serum albumin (BSA). We incubated 1 nmol of ssDNA aptamer, Aci49, previously suspended in 250 µL binding buffer, with the prepared 200 µL of bacterial cultures for 30 min at room temperature. After 30 min the cells were precipitated, and unbound QDs-aptamer were removed along with supernatant by centrifugation at 2000× *g* for 10 min. Cells were washed three times with 1 mL of washing buffer and collected by centrifugation at 2000× *g* for 10 min. 

All cells were then fixed with 4% paraformaldehyde solution. We added 1 mL to the cell pellet and left at room temperature for 20 min. After incubation the cells were washed three times with PBS. The pellet was collected and resuspended in Milli-Q water for further analysis. 

### 2.5. Calibration Curve

To obtain the calibration curve, 8.5 × 10^8^ cells/mL concentration of AB cells were taken. These cells were washed thrice after which five different AB concentrations were prepared at 6%, 12%, 25%, 50% and 100%. Then 20 mg/mL QDs were conjugated to 10 μL of 0.1 mg/mL aptamer and the QD-aptamer conjugate was incubated with all five concentrations of cells in a 96-well plate. 1% BSA was added to avoid nonspecific binding. Fluorescence for each concentration was measured at 320 nm using a plate reader.

### 2.6. Transmission Electron Microscopy

TEM was used to confirm the binding of the QDs-aptamer to the bacteria cells. The analysis was conducted using a 200 KV JEOL 2100F (JEOL, Tokyo, Japan). 

The carbon-coated copper grids were plasma treated for 5 min and 5 µL of the fixed cells were pipetted on the grid then left to dry. After around 20 min, the excess sample was removed using a filter paper and 5 µL of 4% uranyl acetate was placed on the grid and left for 6 min before the grid was washed with ample amounts of DI water to remove the excess salts. The grids were plasma treated for 15 min before loading them into the TEM. All images were analysed using Gatan Microscopy Suite (Ametek., Berwyn, PA, USA). 

### 2.7. Photoluminescence Spectroscopy

Steady-state photoluminescence measurements on InP/ZnS QDs in the range 500–800 nm (excitation wavelength *λ_ex_* = 480 nm) were acquired using the FLS-980 Photoluminescence spectrometer (Edinburgh Instruments Ltd., Livingston, UK). 

### 2.8. Confocal Microscopy

4′,6-diamidino-2-phenylindole (DAPI) was used to stain the nucleus in bacterial cells to confirm the location of the cells. We added 1 mL of DAPI to the previously fixed cell pellet and left at room temperature and in the dark for 20 min. After incubation the cells were washed three times by centrifugation and resuspension of the pellet with PBS. The pellet was collected after centrifugation at 2000× *g* for 10 min and resuspended in Milli-Q water. The cells were then spread on a microscope slide and left to dry in the dark. Unstained cells were also prepared following the same method to avoid any DAPI/QDs emission absorbance overlap. Cells were imaged using an Olympus FV1000 spectral imaging microscope (Olympus Corporation, Tokyo, Japan). The channels parameters were as follows: Dapi channel: excitation $\lambda_{ex}=405\;\mathrm{nm}$ and emission $\lambda_{em}=461\;\mathrm{nm}$. QDs channel: excitation $\lambda_{ex}=473\;\mathrm{nm}$ and emission $\lambda_{em}=520\;\mathrm{nm}$. Bright field: excitation $\lambda_{ex}=405\;\mathrm{nm}$.

## 3. Results and Discussion

The applications of nanotechnology and nanoparticles, such as QDs, to research fields of microbiology remain unadvanced despite their major success in other biological applications. Semiconducting QDs offer attractive features, including high resistance to photodegradation and biocompatibility, which is a requirement for biosensor applications. Herein, we synthesised photoluminescent InP/ZnS QDs following the published procedure by Tessier et al. to produce QDs with oleylamine ligands that are soluble in organic solvents [25]. However, for biological applications in particular bacterial targeting, QDs should be hydrophilic (water-soluble) to be biocompatible. Thus, the ligand exchange procedure adapted by Yong et al. produced mercaptosuccinic acid (MSA) conjugated QDs (QDs-MSA) resulting in a carboxylic acid functionality [26]. The QDs-MSA were water soluble and the terminal carboxylic groups from the MSA allowed for the formation of an amide covalent bond via the amine functionalised aptamers (QDs-aptamer). The ligand exchange was confirmed using Fourier transform infrared spectroscopy (FTIR) in Figure S1, as it showed a C=O stretch at 1700 cm^−1^ and an OH stretch at 3010 cm^−1^ in the QD-MSA sample. Further to this, the sharp C–H stretch at 2900 cm^−1^ was not prominent in the FTIR spectra of the QDs-MSA. In addition, there was a clear migration from the toluene phase (water-insoluble) to the water phase (soluble) which further verifies the ligand exchange. 

As mentioned, there are only a few reports of QDs for bacteria labelling and even fewer using QDs-aptamer as labels. In particular, work by Lan Hee Kim et al. used selenium-based QDs-aptamer to target *P. aeuringosa* with low efficiency [27]. It was hypothesised that the binding affinity was low due to the larger size of the QDs-aptamer probes (20–30 nm). The indium-based QDs used in this work were smaller to avoid any interference from the aptamer binding structure. 

**Figure 1:**
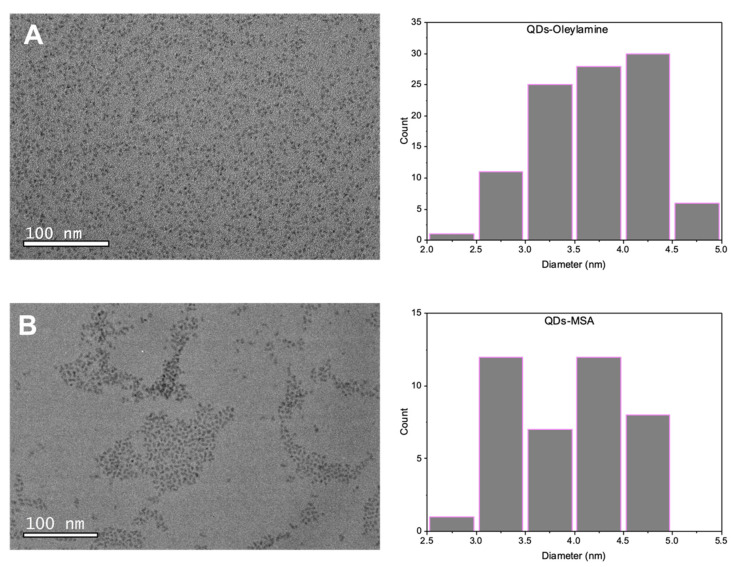
Transmission electron microscopy (TEM) images and size histogram of (**A**) QDs-oleylamine; (**B**) QDs-MSA.

The average size of the QDs was verified using TEM (Figure 1). Both QDs-oleylamine and QDs-MSA showed an average size between 2 and 5 nm. However, this size range makes it unclear whether QDs will hinder the functioning of the attached molecule, herein the aptamer. In addition, their small size may allow them to pass through bacterial cell walls as previously reported [28]. Therefore, a negative control was undertaken using QDs-MSA to determine the uptake of QDs versus the targeting QDs-aptamer moiety. 

Conjugating QDs to targeting ligands such as antibodies and aptamers retains both the photophysical properties of the QDs and the molecular recognition function. Aptamers rival antibodies in both therapeutic and diagnostic applications including biological and bacterial detection [29]. QDs-antibody probes have demonstrated good selectivity for surface bacteria [30,31]. Also, QDs-antibody probes have been shown to be unstable and aggregate in short time periods. In addition, they require lengthy processing steps, and the performance is limited by sample pH and the size of the QDs. QDs-antibody are also costly and can interfere with the bacterial cells growth [32]. Aptamers on the other hand have a strong bioaffinity towards bacteria and can make QDs stable for longer times, even in harsh environments. Unlike antibodies, aptamers can also be denatured (via heat or chemicals) and renatured repeatedly without loss of function [33]. 

**Figure 2:**
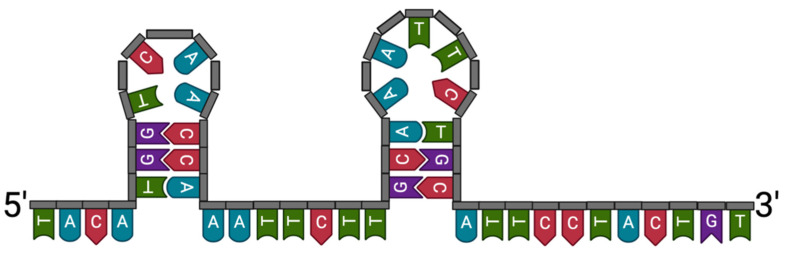
Secondary structure of Aci49 using UNAFold Δ*G* = −4.15 at 21 °C.

The aptamer (Aci49) was selected specifically for *A. baumannii* through a paper published previously by Rasoulinejad et al. [15]. When the target is introduced, aptamers fold into a secondary structure to interact with the bacteria. With aptamer binding, it is known that there are two mechanistic types, (1) lock and key or (2) encapsulation. The encapsulation usually occurs for small molecule detection, whereas the lock and key would be expected for larger targets [34,35,36]. The QDs used here were ~5 nm and were of similar size or slightly smaller compared to the aptamer. Therefore, we can hypothesise the binding mechanism based on the QD conjugate size and target to be a lock and key model. UNAfold was used to determine the secondary form of the selected Aci49 aptamer, (Figure 2) which showed that the aptamer imitates two stem loops. Although it is difficult to postulate how the hairpin loop contributes to the binding of *A. baumannii*, stems and loops and bulges are common features of many DNA aptamers [37]. The authors selected the aptamer through a whole cell SELEX technique in which the *A. baumannii* cells were used as a target to isolate candidate aptamers. Moreover, the aptamer binding functionality is largely dependent on the natural 3D structure of the bacterial cells [38].

The emission spectra were recorded for each type of quantum dot to characterise the photoluminescent properties and to ensure the QDs-aptamers were still functional fluorescent labels. In addition, ligand exchange processes can etch the surface of the quantum dots and sometimes even destroy them. Upon aptamer conjugation the emission spectra of QDs-aptamer showed a mild shift towards the blue when compared to the QDs-MSA (Figure S2). Blue shifts have been reported in QDs before due to oxidation, changes in pH, the presence of divalent cations, and other environmental factors [39]. However, none of the other papers have reported a blue shift after aptamer conjugation, which is usually evidence of the presence of smaller size QDs due to etching. As this process does not remove any ligands to produce smaller QDs, the reason for this blue shift is still unclear. The EDC/NHS coupling could have etched the surface which might alter the QDs size or deform their shape, thereby shifting their emission wavelength.

**Figure 3:**
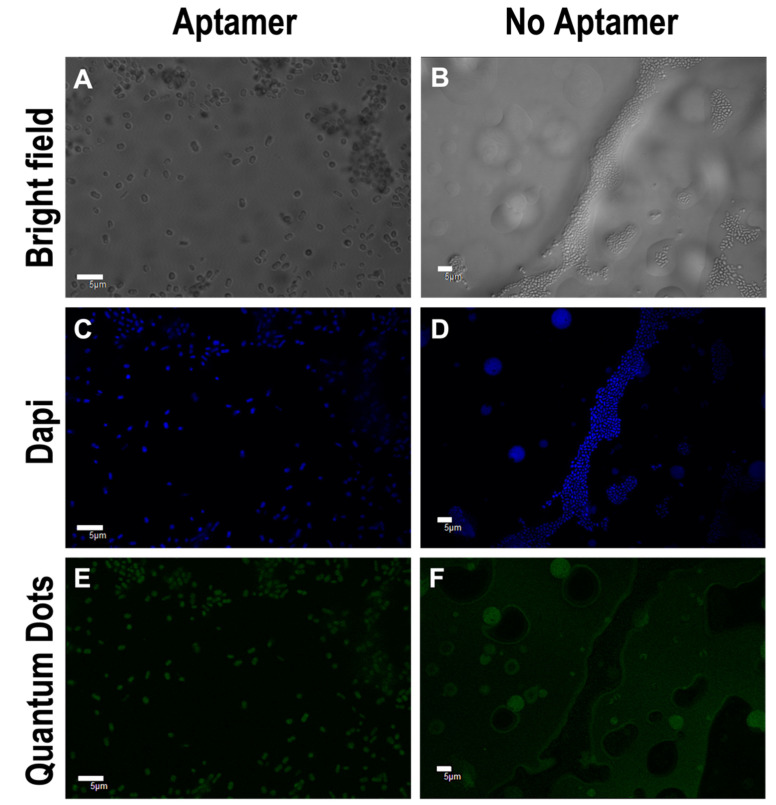
Confocal images of AB9 targeted with QDs-aptamer and QDs-MSA (No Aptamer). Images show cells under bright field (**A**,**B**), Dapi staining the cell’s RNA in blue channel (**C**,**D**), and QDs in the green channel (**E**,**F**). Scale-bar is 5 μm. Dapi $\lambda_{ex}=405\;\mathrm{nm}$, QDs $\lambda_{ex}=473\;\mathrm{nm}$.

Once synthesised, the QD-aptamers needed to be bound to the bacteria to check the binding by confocal microscopy and TEM. At the mid-log phase *A. baumannii* cells were pelleted and washed three times with phosphate-buffered saline (PBS) and a 0.02% tween 20 solution. The QDs-aptamer were incubated with washed bacterial cells for the optimum binding time that also produced intense photoluminescence [27]. Confocal images (Figure 3) verified the specific binding of the QDs-aptamer (green) to bacterial cells and DAPI labelling (blue) of the nucleus confirmed the cell location. QDs-aptamer targeting was compared to the negative control containing unconjugated QDs (QDs-MSA), as shown (Figure 3F) where there is minimal green fluorescence due to high background reflection. The slight green fluorescence is likely due to impurities in the background from QDs that are left in solution. Being water soluble, some of the QDs can pellet down with the bacteria during the washing steps. However, it is obvious that these QDs are in solution rather than labelling the bacterial cells, as per the case of the QDs-aptamer (Figure 3E).

**Figure 4:**
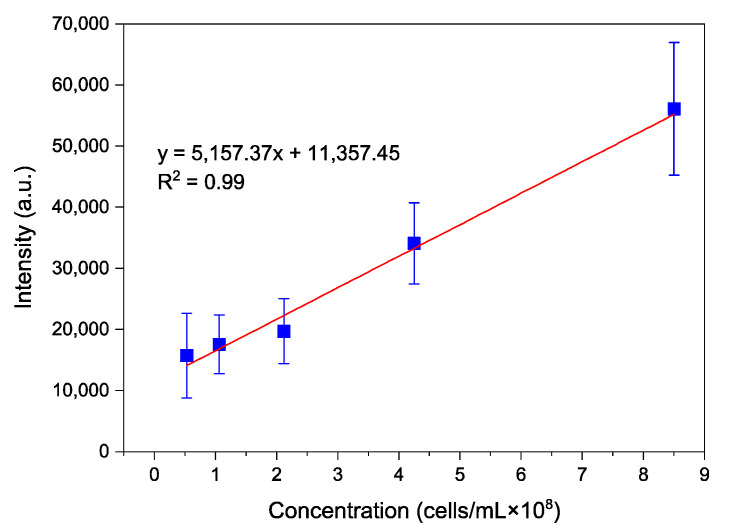
Calibration curve of fluorescence intensity against different concentrations of *A. baumannii*; Error bars represent standard error (*n* = 3). (Excitation wavelength: 320 nm; Emission wavelength: 531 nm).

Since the AB9 was successfully labelled with QD-aptamers and visualised under a confocal microscope, the next step was to verify if the emission intensity could be correlated to the bacteria concentration. In addition, an ELISA plate reader was used to demonstrate the ability to offer a quick and high throughput method. A calibration curve was obtained by recording fluorescence intensities (using $\lambda_{ex}=320\;\mathrm{nm}$) of samples containing QDs-aptamer after incubation (30 min) with *A. baumannii*. Figure 4 illustrates the average intensities with standard errors plotted against increasing concentration (ranging from 0.5 × 10^8^–8.5 × 10^8^ cells/mL). A good linear relationship was obtained demonstrating an increase in photoluminescent intensity in correlation with the concentration of bacteria. The low detection limit was found to be ~0.5 × 10^8^ cells/mL. 

**Figure 5:**
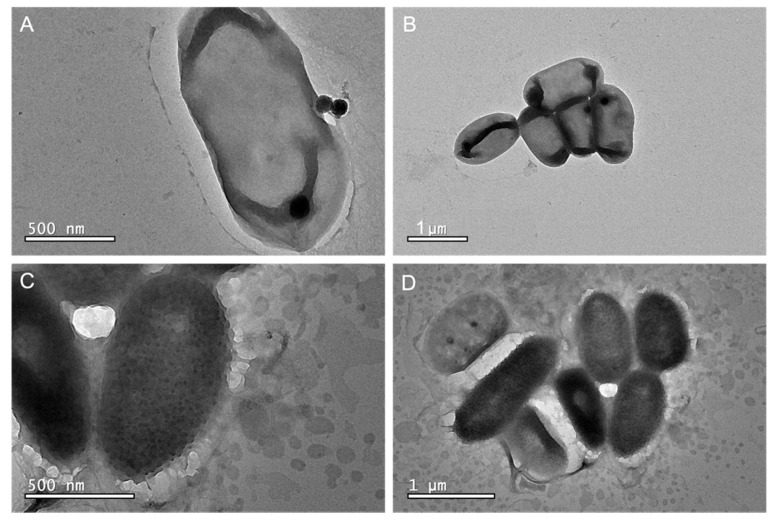
TEM images of uranyl acetate stained AB9 cells showing clear transparent membrane (**A**,**B**). QDs-Aptamer targeted bacteria show the QDs covering the membrane of the cells (**C**,**D**).

To gain information on the morphology of the bacteria and the location of the QDs, TEM was used. TEM images of bacteria cells complimented the results thus far, further verifying the presence of QDs-aptamer on the bacteria cells. Dark spots thought to be QDs attached to the *A. baumannii* cells through the aptamer can be seen (Figure 5C,D), compared to the clear transparent membrane of the cells (no bound QDs) in the negative control (Figure 5A,B). The QDs however seem to be bigger in size compared to the measured size before cell labelling. It is hypothesised that the QDs are potentially aggregating, and the dark spots could be multiple QDs rather than just one. Alternatively, it could be an artefact of the large, dehydrated bacteria causing the QDs to appear bigger. This could mean that the aptamers are being attracted towards the same spot on the bacteria membrane. Alternatively, the uranyl acetate would stain the aptamer which makes the QDs attached to the aptamer appear to be darker and larger in diameter [40].

**Figure 6:**
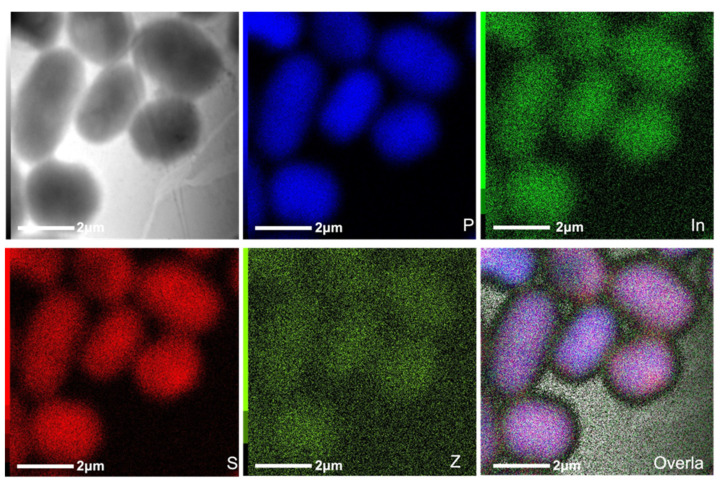
Scanning transmission electron microscopy (STEM) images of AB9 targeted with aptamer-QDs and stained with uranyl acetate with an elemental map for phosphorous (blue), sulphur (red), indium (bright green) and zinc (dark green). Scale-bar 2 μm.

Scanning TEM (STEM) maps were run for elemental composition and location. As shown, phosphorus (Figure 6, blue) is highly concentrated in the location of the bacterial cells. However, energy-dispersive X-ray spectroscopy (EDS) spectra of *A. baumannii* show a significant phosphorus peak, which is unsurprising as phosphorus is naturally present in living organisms (Figure S3). The InP/ZnS QDs also contain phosphorus, and it is therefore impossible to attribute this in the STEM map to the QDs and/or the bacteria itself. However, no traces of indium or zinc are seen on the EDS spectra of the bacteria (Figure S3), whereas a clear concentration of these elements on the elemental map (Figure 6, bright green and dark green) confirms that QDs-aptamer did target this bacterium.

The same experiments were run using another strain of *A. baumannii* (AB11). The results were complimentary, confirming that the QDs-aptamer can target different strains of *A. baumannii* (Figure S4). This is an important key in the detection of bacteria, as strains of *A. baumannii* possess a high mutation rate [41]. It is important that the system can target multiple strains as per the case of PCR detection. For this, previous knowledge of the strain’s sequence or domain is required, limiting the detection of new strains that need to be added to the database [42,43]. 

To study the specific binding of the aptamer to other bacteria species, a negative control was carried out using *P. aeruginosa*. The *P. aeruginosa* cells were targeted with the same QDs-aptamer using the same conditions. Confocal images in Figure S5 show no fluorescence from the QDs-aptamer. TEM images of *P. aeruginosa* with QDs-aptamer from Figure S6 show a clear transparent membrane with no trace of QDs on the surface of cells to further confirm the specificity of the QDs-aptamer. This provides some insight into the binding of the aptamer to the bacteria. The QD-aptamer conjugate attaches to the surface of AB9 bacteria but not to the surface of *P. aeruginosa* cells. This means that the aptamer targets a moiety that is specific to the AB9 bacteria, such as a cell membrane protein. For example, lipopolysaccharides are present on the outer surface membranes of almost all Gram-negative bacteria. As the QD-Aptamer is not attached to the surface of other bacteria (such as *P. aeruginosa*), we can rule this out as a target. More studies would be needed to find the exact target, but such studies are known to be difficult.

## 4. Conclusions

In conclusion, InP/ZnS QDs were successfully synthesised and conjugated to the chosen aptamer to label *A. baumannii*. Photoluminescent labelling of the bacteria was verified using confocal microscopy and TEM. The QDs-aptamer bioconjugates showed specificity to the *A. baumannii* cells and no binding to other bacteria, such as *P. aeruguinosa* cells. After incubation, photoluminescent labelling was observed with QDs-aptamer bound to the surface of *A. baumannii* as shown via confocal microscopy, TEM and photoluminescent spectroscopy. This easy and fast labelling method can be applied in the detection of *A. baumannii* from different environments such as wastewater treatment plants and activated sludge.

In addition, microfluidics technology has been widely used in sample preparation, separation, and detection process. This simple, automatic, rapid, and portable system is rarely used in the detection of pathogens. Combining our QDs-aptamer bioconjugates with microfluidics may lead to a fast, sensitive, and portable detection platform for pathogenic microorganisms. Such a system can achieve the truly automated real-time testing, that can be translated to other detection applications.

## Data Availability

Data presented in this article is available on request from the corresponding author.

## Supplementary Materials

The following are available online at https://www.mdpi.com/article/10.3390/nano11123317/s1. Figure S1: Fourier transform infrared spectroscopy (FTIR) spectra of the InP/ZnS–oleylamine and InP/ZnS–MSA. Figure S2: Normalized emission spectra of QDs-MSA and QDs-aptamer. Figure S3: EDS spectrum of *A. baumannii* stained with uranyl acetate. Figure S4: Confocal images of *A. baumannii* (AB11) targeted with QDs-aptamer and QDs-MSA. Figure S5: Confocal images of DAPI stained *P. aeruginosa* mixed with QDs-aptamer. Figure S6: TEM images of Uranyl acetate-stained *P. aeruginosa* cells with QDs-aptamer.

## References

[1] Zhao X., Hilliard L.R., Mechery S.J., Wang Y., Bagwe R.P., Jin S., Tan W. (2004). A rapid bioassay for single bacterial cell quantitation using bioconjugated nanoparticles. Proc. Natl. Acad. Sci. USA.

[2] World Health Organization Guidelines for the Prevention and Control of Carbapenem-Resistant Enterobacteriaceae, Acinetobacter Baumannii and Pseudomonas Aeruginosa in Health Care Facilities. https://www.who.int/publications/i/item/guidelines-for-the-prevention-and-control-of-carbapenem-resistant-enterobacteriaceae-acinetobacter-baumannii-and-pseudomonas-aeruginosa-in-health-care-facilities.

[3] Fournier P.E., Richet H. (2006). The epidemiology and control of Acinetobacter baumannii in health care facilities. Clin. Infect. Dis..

[4] Anstey N.M., Currie B.J., Withnall K.M. (1992). Community-acquired acinetobacter pneumonia in the Northern Territory of Australia. Clin. Infect. Dis..

[5] Chu Y.W., Leung C.M., Houang E.T.S., Ng K.C., Leung C.B., Leung H.Y., Cheng A.F.B. (1999). Skin carriage of acinetobacters in Hong Kong. J. Clin. Microbiol..

[6] Wenzler E., Goff D.A., Mangino J.E., Reed E.E., Wehr A., Bauer K.A. (2016). Impact of rapid identification of Acinetobacter Baumannii via matrix-assisted laser desorption ionization time-of-flight mass spectrometry combined with antimicrobial stewardship in patients with pneumonia and/or bacteremia. Diagn. Microbiol. Infect. Dis..

[7] Kempf M., Rolain J.M. (2012). Emergence of resistance to carbapenems in Acinetobacter baumannii in Europe: Clinical impact and therapeutic options. Int. J. Antimicrob. Agents.

[8] Kempf M., Abdissa A., Diatta G., Trape J.F., Angelakis E., Mediannikov O., La Scola B., Raoult D. (2012). Detection of Acinetobacter baumannii in human head and body lice from Ethiopia and identification of new genotypes. Int. J. Infect. Dis..

[9] Towner K.J. (2009). Acinetobacter: An old friend, but a new enemy. J. Hosp. Infect..

[10] Garnacho-Montero J., Dimopoulos G., Poulakou G., Akova M., Cisneros J.M., De Waele J., Petrosillo N., Seifert H., Timsit J.F., Vila J. (2015). Task force on management and prevention of Acinetobacter baumannii infections in the ICU. Intensive Care Med..

[11] Soo P.C., Tseng C.C., Ling S.R., Liou M.L., Liu C.C., Chao H.J., Lin T.Y., Chang K.C. (2013). Rapid and sensitive detection of Acinetobacter baumannii using loop-mediated isothermal amplification. J. Microbiol. Methods.

[12] Turton J.F., Woodford N., Glover J., Yarde S., Kaufmann M.E., Pitt T.L. (2006). Identification of Acinetobacter baumannii by detection of the bla OXA-51-like carbapenemase gene intrinsic to this species. J. Clin. Microbiol..

[13] Liu H.Y., Hopping G.C., Vaidyanathan U., Ronquillo Y.C., Hoopes P.C., Moshirfar M. (2019). Polymerase Chain Reaction and Its Application in the Diagnosis of Infectious Keratitis. Med. Hypothesis Discov. Innov. Ophthalmol. J..

[14] Acquah C., Danquah M.K., Yon J.L.S., Sidhu A., Ongkudon C.M. (2015). A review on immobilised aptamers for high throughput biomolecular detection and screening. Anal. Chim. Acta.

[15] Rasoulinejad S., Gargari S.L.M. (2016). Aptamer-nanobody based ELASA for specific detection of Acinetobacter baumannii isolates. J. Biotechnol..

[16] Dunn M.R., Jimenez R.M., Chaput J.C. (2017). Analysis of aptamer discovery and technology. Nat. Rev. Chem..

[17] Ikanovic M., Rudzinski W.E., Bruno J.G., Allman A., Carrillo M.P., Dwarakanath S., Bhahdigadi S., Rao P., Kiel J.L., Andrews C.J. (2007). Fluorescence assay based on aptamer-quantum dot binding to bacillus thuringiensis spores. J. Fluoresc..

[18] Bilan R., Nabiev I., Sukhanova A. (2016). Quantum Dot-Based Nanotools for Bioimaging, Diagnostics, and Drug Delivery. ChemBioChem.

[19] Su C.H., Tsai M.H., Lin C.Y., Ma Y.D., Wang C.H., Chung Y.D., Lee G.B. (2020). Dual aptamer assay for detection of Acinetobacter baumannii on an electromagnetically-driven microfluidic platform. Biosens. Bioelectron..

[20] Wen L., Qiu L., Wu Y., Hu X., Zhang X. (2017). Aptamer-modified semiconductor quantum dots for biosensing applications. Sensors.

[21] Ayupova D., Dobhal G., Laufersky G., Nann T., Goreham R.V. (2019). An in vitro investigation of cytotoxic effects of InP/ZnS quantum dots with different surface chemistries. Nanomaterials.

[22] Brunetti V., Chibli H., Fiammengo R., Galeone A., Malvindi M.A., Vecchio G., Cingolani R., Nadeau J.L., Pompa P.P. (2013). InP/ZnS as a safer alternative to CdSe/ZnS core/shell quantum dots: In vitro and in vivo toxicity assessment. Nanoscale.

[23] Tran P.T., Goldman E.R., Anderson G.P., Mauro J.M., Mattoussi H. (2002). Use of Luminescent CdSe-ZnS Nanocrystal Bioconjugates in Quantum Dot-Based Nanosensors. Phys. Status Solidi (B).

[24] Kloepfer J.A., Mielke R.E., Wong M.S., Nealson K.H., Stucky G., Nadeau J.L. (2003). Quantum dots as strain- and metabolism-specific microbiological labels. Appl. Environ. Microbiol..

[25] Tessier M.D., Dupont D., De Nolf K., De Roo J., Hens Z. (2015). Economic and Size-Tunable Synthesis of InP/ZnE (E = S, Se) Colloidal Quantum Dots. Chem. Mater..

[26] Yong K.T., Ding H., Roy I., Law W.C., Bergey E.J., Maitra A., Prasad P.N. (2009). Imaging pancreatic cancer using bioconjugated inp quantum dots. ACS Nano.

[27] Kim L.H., Yu H.-W., Kim Y.-H., Kim I.S., Jang A. (2013). Potential of Fluorophore Labeled Aptamers for Pseudomonas aeruginosa Detection in Drinking Water. J. Korean Soc. Appl. Biol. Chem..

[28] Kloepfer J.A., Mielke R.E., Nadeau J.L. (2005). Uptake of CdSe and CdSe/ZnS quantum dots into bacteria via purine-dependent mechanisms. Appl. Environ. Microbiol..

[29] Bruno J.G. (2014). Application of DNA aptamers and quantum dots to lateral flow test strips for detection of foodborne pathogens with improved sensitivity versus colloidal gold. Pathogens.

[30] Wang Y., Yan X.P. (2013). Fabrication of vascular endothelial growth factor antibody bioconjugated ultrasmall near-infrared fluorescent Ag2S quantum dots for targeted cancer imaging in vivo. Chem. Commun..

[31] Yang L., Li Y. (2006). Simultaneous detection of Escherichia coli O157:H7 and Salmonella Typhimurium using quantum dots as fluorescence labels. Analyst.

[32] Abdelhamid H.N., Wu H.F. (2013). Probing the interactions of chitosan capped CdS quantum dots with pathogenic bacteria and their biosensing application. J. Mater. Chem. B.

[33] Jayasena S.D. (1999). Aptamers: An emerging class of molecules that rival antibodies in diagnostics. Clin. Chem..

[34] Lakhin A.V., Tarantul V.Z., Gening L.V. (2013). Aptamers: Problems, solutions and prospects. Acta Nat..

[35] Song K.M., Jeong E., Jeon W., Cho M., Ban C. (2012). Aptasensor for ampicillin using gold nanoparticle based dual fluorescence-colorimetric methods. Anal. Bioanal. Chem..

[36] Shannon Pendergrast P., Nicholas Marsh H., Grate D., Healy J.M., Stanton M. (2005). Nucleic acid aptamers for target validation and therapeutic applications. J. Biomol. Tech..

[37] Mann D., Reinemann C., Stoltenburg R., Strehlitz B. (2005). In vitro selection of DNA aptamers binding ethanolamine. Biochem. Biophys. Res. Commun..

[38] Dickey D.D., Giangrande P.H. (2016). Oligonucleotide aptamers: A next-generation technology for the capture and detection of circulating tumor cells. Methods.

[39] Sark W.V., Frederix P.L., Bol A.A., Gerritsen H.C., Meijerink A. (2002). Blueing, Bleaching, and Blinking of Single CdSe/ZnS Quantum Dots. J. Phys. Chem. A.

[40] Stoeckenius W. (1961). Electron microscopy of DNA molecules “stained” with heavy metal salts. J. Biophys. Biochem. Cytol..

[41] Karami-Zarandi M., Douraghi M., Vaziri B., Adibhesami H., Rahbar M., Yaseri M. (2017). Variable spontaneous mutation rate in clinical strains of multidrug-resistant Acinetobacter baumannii and differentially expressed proteins in a hypermutator strain. Mutat. Res.-Fundam. Mol. Mech. Mutagen..

[42] Nakano K., Nomura R., Shimizu N., Nakagawa I., Hamada S., Ooshima T. (2004). Development of a PCR method for rapid identification of new Streptococcus mutans serotype k strains. J. Clin. Microbiol..

[43] Van Helden P.D. (1998). Bacterial genetics and strain variation. Novartis Found. Symp..

