# Supplementary material for: Aptamer Conjugated Indium Phosphide Quantum Dots with a Zinc Sulphide Shell as Photoluminescent Labels for Acinetobacter baumannii
Source: Nanomaterials (Basel). 2021 Dec 7;11(12):3317. doi: 10.3390/nano11123317 (PMC8703687; doi:10.3390/nano11123317)
Supplement: Supplementary file 1 [file nanomaterials-11-03317-s001.zip › nanomaterials-1478244-supplementary.pdf]

nanomaterials

MDPI

Supplementary Material

Aptamer Conjugated Indium Phosphide Quantum Dots with a Zinc Sulfide Shell as Photoluminescent Labels for *Acinetobacter baumannii*

Zeineb Ayed [1,2], Shiana Malhotra [3], Garima Dobhal [3] and Renee V. Goreham [3,*]

1 The MacDiarmid Institute, Wellington 6012, New Zealand; zeineb.ayed@vuw.ac.nz
2 School of Chemical Physical Sciences, Victoria University of Wellington, Kelburn, Wellington 6012, New Zealand
3 School of Mathematical and Physical Sciences, University of Newcastle, Newcastle NSW 2308, Australia; shiana.malhotra@uon.edu.au (S.M.); gsd534@newcastle.edu.au (G.D.)
* Correspondence: renee.goreham@newcastle.edu.au; Tel.: +61-086-7066

Citation: Ayed, Z.; Malhotra, S.; Dobhal, G.; Goreham, R.V. Aptamer Conjugated Indium Phosphide Quantum Dots with a Zinc Sulfide Shell as Photoluminescent Labels for *Acinetobacter baumannii*. *Nanomaterials* **2021**, *11*, 3317. https://doi.org/10.3390/nano11123317

Received: 9 November 2021
Accepted: 3 Decembrie 2021
Published: 7 Decembrie 2021

Publisher's Note: MDPI stays neutral with regard to jurisdictional claims in published maps and institutional affiliations.

Copyright: © 2021 by the authors. Submitted for possible open access publication under the terms and conditions of the Creative Commons Attribution (CC BY) license (https://creativecommons.org/licenses/by/4.0/).

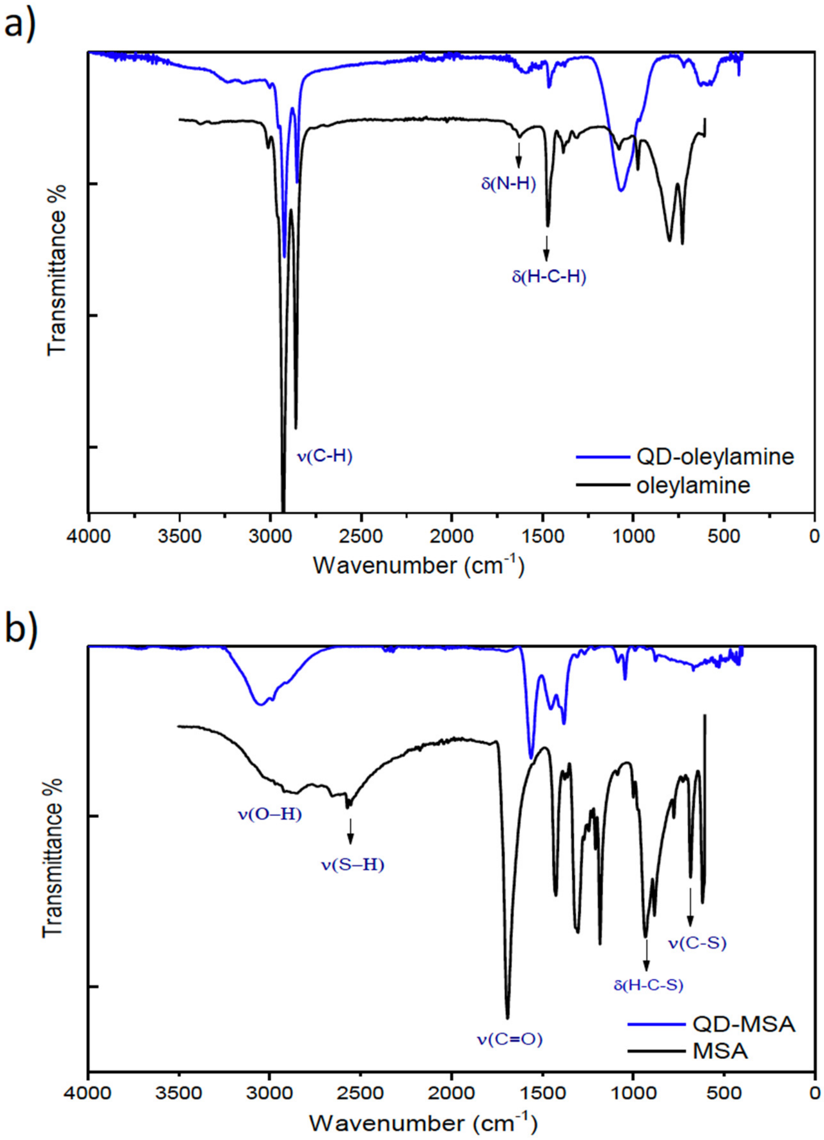

Figure S1. Fourier transform infrared spectroscopy (FTIR) spectra of the InP/Zns QDs–oleylamine (**a**), and InP/ZnS QDs–MSA (**b**).

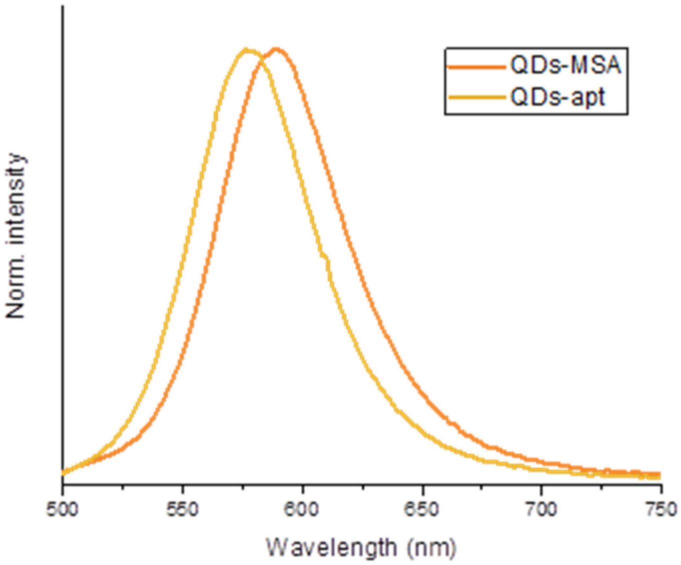

Figure S2. Normalized emission spectra of QDs-MSA and QDs-Aptamer. Excitation λ_{ex} = 480 nm.

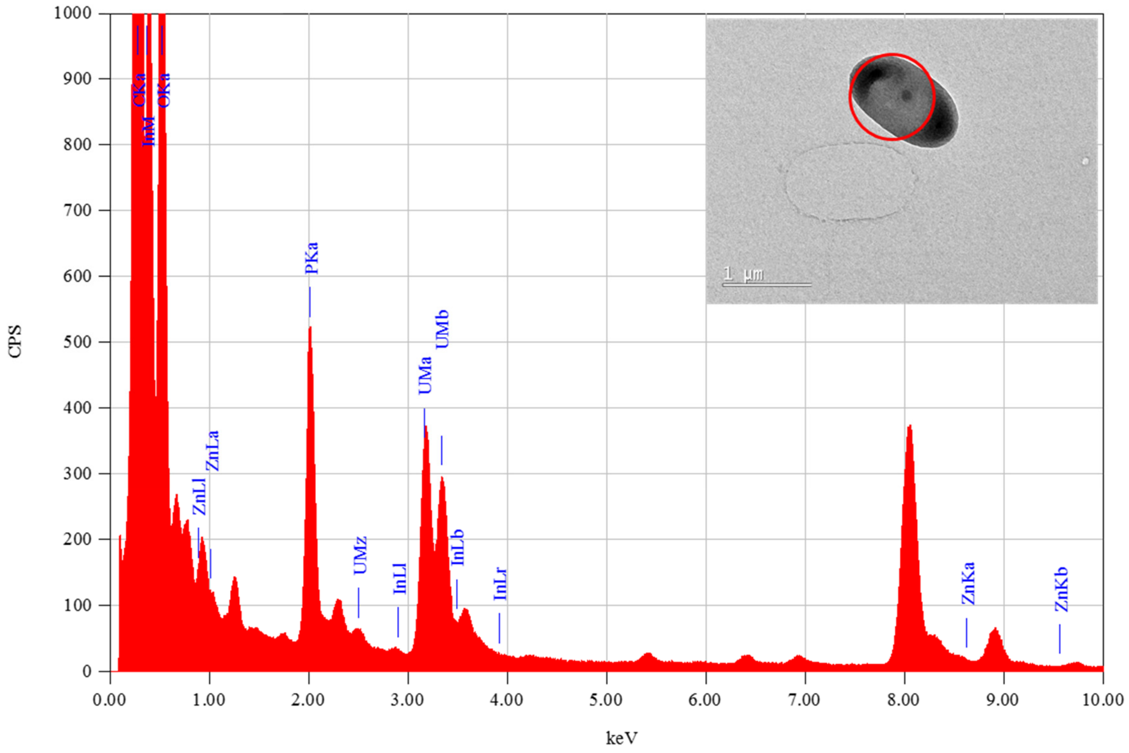

Figure S3. EDS spectrum of *A. baumannii* stained with uranyl acetate showing high phosphorous and uranyl peaks and no traces of Zinc or Indium. Inset is a TEM image of the bacterium and the location of the EDS. Unit: CPS: Count/sec.

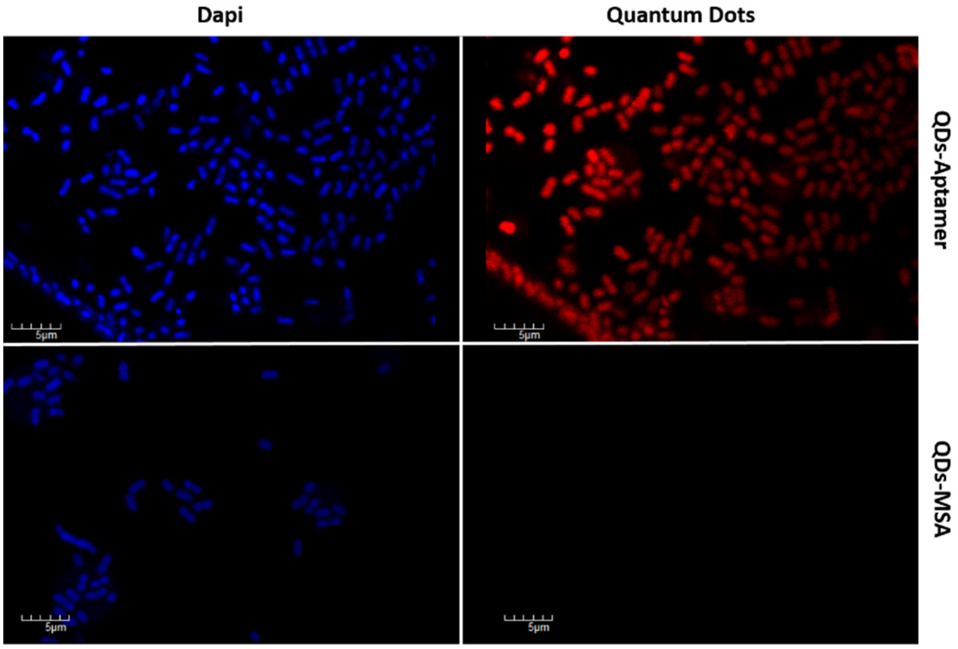

Figure S4. Confocal images of *A. baumannii* (AB11) targeted with QDs-aptamer (top) and QDs-MSA (bottom). Images show DAPI staining the cells RNA with blue, QDs staining the cells with red. Scale-bar is 5 μm.

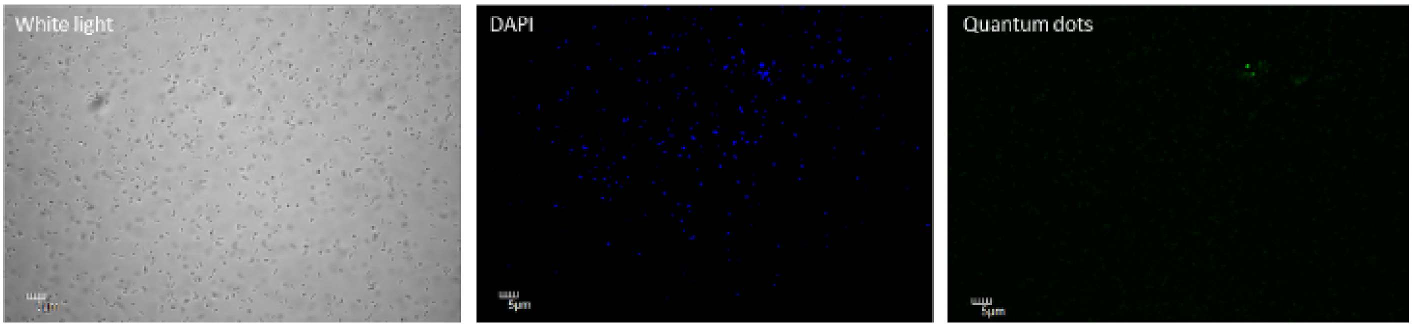

Figure S5. Confocal images of DAPI stained *P. aeruginosa* mixed with QDs-aptamer. Scale-bar is 5 μm.

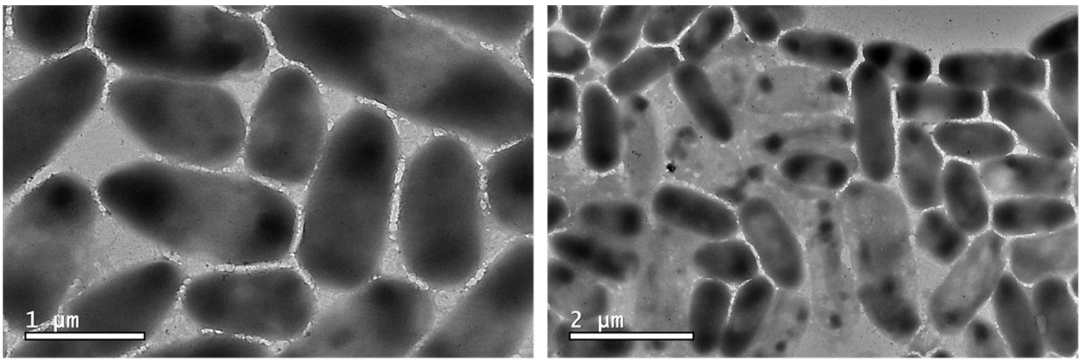

Figure S6. TEM images of Uranyl acetate-stained P. aeruginosa cells with QDs-Aptamer showing clear transparent membrane.
